# Physical and *in silico *approaches identify DNA-PK in a Tax DNA-damage response interactome

**DOI:** 10.1186/1742-4690-5-92

**Published:** 2008-10-15

**Authors:** Emad Ramadan, Michael Ward, Xin Guo, Sarah S Durkin, Adam Sawyer, Marcelo Vilela, Christopher Osgood, Alex Pothen, Oliver J Semmes

**Affiliations:** 1 Department of Computer Science, Old Dominion University, Norfolk, VA, USA; 2 George L. Wright Center for Biomedical Proteomics, Eastern Virginia Medical School, Norfolk, VA, USA; 3 Department of Microbiology and Molecular Cell Biology, Eastern Virginia Medical School, Norfolk, VA, USA; 4 Laboratorio do Cancer, Univeridade Federal de Vicosa, Minas Gerais, Brazil; 5 Department of Biology, Old Dominion University, Norfolk, VA, USA; 6 Department of Computer Sciences and Computing Research Institute, Purdue University, West Lafayette IN, USA; 7 Department of Exploratory Biology, Pfizer Global Research and Development, La Jolla, CA, USA

## Abstract

**Background:**

We have initiated an effort to exhaustively map interactions between HTLV-1 Tax and host cellular proteins. The resulting Tax interactome will have significant utility toward defining new and understanding known activities of this important viral protein. In addition, the completion of a full Tax interactome will also help shed light upon the functional consequences of these myriad Tax activities. The physical mapping process involved the affinity isolation of Tax complexes followed by sequence identification using tandem mass spectrometry. To date we have mapped 250 cellular components within this interactome. Here we present our approach to prioritizing these interactions via an *in silico *culling process.

**Results:**

We first constructed an *in silico *Tax interactome comprised of 46 literature-confirmed protein-protein interactions. This number was then reduced to four Tax-interactions suspected to play a role in DNA damage response (Rad51, TOP1, Chk2, 53BP1). The first-neighbor and second-neighbor interactions of these four proteins were assembled from available human protein interaction databases. Through an analysis of betweenness and closeness centrality measures, and numbers of interactions, we ranked proteins in the first neighborhood. When this rank list was compared to the list of physical Tax-binding proteins, DNA-PK was the highest ranked protein common to both lists. An overlapping clustering of the Tax-specific second-neighborhood protein network showed DNA-PK to be one of three bridge proteins that link multiple clusters in the DNA damage response network.

**Conclusion:**

The interaction of Tax with DNA-PK represents an important biological paradigm as suggested via consensus findings *in vivo *and *in silico*. We present this methodology as an approach to discovery and as a means of validating components of a consensus Tax interactome.

## Background

Human T-cell Leukemia Virus type 1(HTLV-1) is the causative agent of Adult T-cell Leukemia (ATL), HTLV-1 Associated Myelopathy/Tropical Spastic Paraparesis (HAM/TSP) as well as other subneoplastic conditions [1-5]. Although the development of ATL is the culmination of complex events, it appears that the viral oncogene product, Tax, may provide the impetus for the transformation process. This protein has been studied extensively since 1982 when Tax was discovered to be a transactivator of the cognate viral promoter [6]. Since that time many activities and subsequent functions have been assigned to the Tax protein [7-9]. The critical importance of this protein to human disease makes it a fascinating protein as a research target; however, the result of such focused research efforts has been thousands of articles and a healthy dose of controversy. These qualities also make Tax an ideal candidate for the development of a complete list of interacting proteins as an effort to define potential protein functions.

There have been a number of published accounts of cellular proteins that bind to Tax. For example, Jin et al described the binding of Tax to MAD1 as a result of a comprehensive yeast two-hybrid approach [10]. Immunoprecipitation and western analysis has been used to identify specific Tax-protein interactions, for example IKKγ [11,12], CRM1 [13], Dlg1 [14] and components of the APC [15,16]. Recently, Kashanchi and co-workers conducted a major effort using 2D gel separation followed by MALDI-MS to identify a 32-member Tax interactome [17]. A combined listing of Tax binding proteins with accompanying literature citations can be found by visiting the publicly accessible Tax website .

As data accumulates regarding Tax-protein interactions, a system for analysis and validation of these interactions is needed. This is especially true given the exponential increase in technical ability to identify protein-protein interactions, compounded by the inherent increases in false-positives (protein-protein interactions of no functional consequence). We describe a two-pronged approach for identification and selection of functionally significant Tax-protein interactions. The study begins with the construction of a comprehensive physical interactome using affinity isolation of Tax complexes coupled to MS/MS analysis. Next, we utilized knowledge gained in existing literature that defined a physical interaction between Tax and a cellular protein, to comprise an *in silico *Tax interactome. This interactome was then restricted to proteins with a putative role in DNA repair response. The final steps expanded the *in silico *interactions into a nearest neighbor network to identify groups of proteins with greatest functional impact to DNA repair response. Our analysis identified DNA-PK as a top candidate protein for further analysis into the mechanism of action for Tax-induced defects in the cellular DNA damage repair response.

## Results

### Assimilation of an interaction database for Tax

We conducted a manual literature search for articles with reference to "Tax Interaction". This list of research articles was then limited to those that could be manually confirmed as containing evidence of Tax binding via physical interaction. The manual filtering resulted in a confirmed list of 67 proteins (see Table 1). As we have alluded to earlier, Tax has many putative functions but for this exercise we have limited our analysis to the DNA damage repair response. Thus, we asked which of these known protein interactions has a known function that would potentially impact the cellular DNA repair response process. Our analysis suggested a starting point of four confirmed Tax-binding proteins; Rad51, TOP1, Chk2, and 53BP1.

**Table 1:** Tax interacting proteins

**Tax interacting protein**	**Evidence for interaction**	**Alternate names**	**Reference**
PCAF	GST pulldown; co-IP	p300/CBP-associated factor	Jiang H, MCB 1999 19(12):8136-45
PSAP	GST pulldown	Sap-1	Shuh M, J. Virol 2000 74(23):11394
ELK1	GST pulldown	ETS family	Shuh M, J. Virol 2000 74(23):11394
SRF	GST pulldown	serum response factor	Shuh M, J. Virol 2000 74(23):11394
SUV39H1	GST pulldown; co-IP	KMT1A	Kamoi K, Retrovirology 2006 3:5
ATF4	yeast two hybrid; GST pulldown	TAXREB67, CREB-2	Reddy TR, Oncogene 1997 14(23):2785
MSX2	co-IP	CRS2, FPP, HOX8, MSH, PFM	Twizere JC, JBC 2005 280(33):29804
ZFP36	GST pulldown; co-IP; Colocalization	tristetraprolin, TTP, NUP475	Twizere JC, JNCI 2003 95(24):1846
CREBBP	GST pulldown; co-IP; Colocalization	CREB binding protein, CBP	Bex F, MCB 1998 18(4):2392
p300	GST pulldown; co-IP; colocalization	p300, KAT3B	Bex F, MCB 1998 18(4):2392
MAP3K1	co-IP	MEKK, MAPKKK1	Yin MJ, Cell 1998 93(5):875
ACTL6A	co-IP	BAF53, Arp4, INO80K	Wu K, JBC 2004 279(1):495
SMARCE1	co-IP	BAF57, SWI/SNF related	Wu K, JBC 2004 279(1):495
SMARCC1	co-IP	BAF155, SWI/SNF related	Wu K, JBC 2004 279(1):495
BRG1	co-IP	SMARCA4, SWI/SNF related	Wu K, JBC 2004 279(1):495
RAD51	co-IP	BRCC5	Wu K, JBC 2004 279(1):495
RAG2	co-IP		Wu K, JBC 2004 279(1):495
Actin	co-IP	ACTA	Wu K, JBC 2004 279(1):495
CDK2	co-IP		Wu K, JBC 2004 279(1):495
CDC42	co-IP	G25K	Wu K, JBC 2004 279(1):495
RHOA	co-IP		Wu K, JBC 2004 279(1):495
RAC1	co-IP	TC-25, p21-Rac1	Wu K, JBC 2004 279(1):495
GSN	co-IP	gelsolin	Wu K, JBC 2004 279(1):495
RASA2	co-IP	GAP1M	Wu K, JBC 2004 279(1):495
TAX1BP1	yeast two hybrid, GST pulldown, Co-localisation	TXBP151, CALCOCO3	Reddy TR, PNAS 95(2): 702
CHEK2	Co-IP, co-localization	CDS1, CHK2	Haoudi A, JBC 2003 278(39):37736
RB1	GST pulldown	retinoblastoma 1	Kehn K, Oncogene 2005 24(4):525
CCND2	in vitro binding	Cyclin D2	Fraedrich K, Retrovirology 2005 2:54
CDK4	in vitro binding, mammalian two hybrid	PSK-J3	Fraedrich K, Retrovirology 2005 2:54
IKBKB	co-IP	IKK-beta, IKK2, FKBIKB	Harhaj EW, JBC 274(33):22911
IKBKG	co-IP	IKK-gamma, NEMO, FIP3	Harhaj EW, JBC 274(33):22911
CREB1	co-IP		Zhao LJ, PNAS 89(15):7070
MAD1	yeast two hybrid	TXBP181, MAD1L1, PIG9	Jin DY, Cell 93(1):81
CDC27	co-IP	APC3	Liu B, PNAS 2005 102(1):63
CDC20	co-IP	p55CDC, CDC20A	Liu B, PNAS 2005 102(1):63
RELA	co-IP	NFKB3; p65	Lacoste, Leukemia 1994 8 Suppl 1:S71
NFYB	yeast two hybrid; GST pulldown; co-IP	CBF-A, HAP3	Pise-Masison CA, MCB 1997 17(3):1236
NFKB1	co-IP	KBF1, p105	Beraud C, MCB 1994 14(2):1374
RAN	GST pulldown; co-IP; Colocalization	ARA24, TC4, Gsp1	Peloponese JM, PNAS 2005 102(52):18974
RANBP1	GST pulldown; co-IP; Colocalization	HTF9A	Peloponese JM, PNAS 2005 102(52):18974
CEBPB	GST pulldown	LAP, CRP2, NFIL6, TCF5	Tsukada J, Blood 1997 90(8):3142
TBP	GST pulldown	TFIID	Caron C, EMBO J 1993 12(11):4269
TAF11	GST pulldown; co-IP	TAF(II)28, RNA polymerase II	Caron C, PNAS 1997 94(8):3662
HDAC1	co-IP, GST pulldown	HD1, GON-10	Ego T, Oncogene 2002 21(47):7241
ATF5	yeast two hybrid, co-IP	ATFx	Forgacs E, J Virol 2005 79(11):6932
NRF1	GST pulldown	EWG, ALPHA-PAL	Moriuchi M, AIDS Res Hum Retroviruses 1999 15(9):821
CDK9	GST pulldown; co-IP	PITALRE, C-2k, TAK	Zhou M, J Virol 2006 80(10):4781
MAGI3	co-IP; colocalization		Ohashi M, Virology 2004 320(1):52
DNAJA3	GST pulldown;	TID1, hTid-1	Cheng H, Curr Biol 2001 11(22):1771
HSPA2	GST pulldown; Colocalization	HSP70-2	Cheng H, Curr Biol 2001 11(22):1771
HSPA1B	GST pulldown; Colocalization	HSP70-2	Cheng H, Curr Biol 2001 11(22):1771
TOP1	yeast two hybrid; co-IP	DNA topoisomerase 1	Suzuki T, Virology 2000 270(2):291
CHUK	co-IP	IKK-alpha, IKK1, IKKA	Chu ZL, JBC 1999 274(22): 15297
SPI1	GST pulldown	p16INK4A; MTS1, p19ARF	Tsukada J, Blood 1997 90(8):3142
CDKN2A	GST pulldown; co-IP	p16INK4A; MTS1, p19ARF	Suzuki T, EMBO J 1996 15(7):1607
GTF2A1	yeast two-hybrid; GST-pulldown; co-IP	TFIIA	Clemens KE, MCB 1996 16(9):465
CDKN1A	co-IP	p21CIP1/WAF1, CAP20	Haller K, MCB 2002 22(10):3327
NFKB2	co-IP	LYT-10	Murakami T, Virology 1995 206(2):1066
VAC14	co-IP	TAX1BP2; TRX	Mireskandari A, BBA 1996 1306(1):9
GPS2	yeast two hybrid; GST pulldown	TXBP31	Jin DY, JBC 1997 272(41):25816
CCND3	co-IP	Cyclin D3	Haller K, MCB 2002 22(10):3327
PSMB4	yeast two hybrid; co-IP	HN3	Haller K, MCB 2002 22(10):3327
PSMA4	yeast two hybrid; co-IP	HC9; PSC9	Rousset R, Nature 1996 381(6580):328
CARM1	GST pulldown; co-IP; Colocalization	PRMT4	Jeong SJ, J Virol 2006 80(20):10036
GNB2	yeast two hybrid; co-IP; Colocalization	transducin beta chain 2	Twizere JC, Blood 2007 109(3):1051
GNB5	co-IP; colocalization	GB5	Twizere JC, Blood 2007 109(3):1051
GNB1	co-IP; colocalization	transducin beta chain 1	Twizere JC, Blood 2007 109(3):1051
IL16	co-IP, colocalization	LCF	Wilson KC, Virology 2003 306(1):60
PPP2CA	co-IP, GST pulldown	PP2A catalytic subunit	Fu DX, JBC 2003 278(3):1487
MAP3K14	co-IP	NIK	Xiao G, EMBO J 2001 20(10):6805
TP53BP1	co-IP, colocalization	53BP1, p202	Haoudi A, JBC 2003 278(39):37736

### Construction of a physical Tax interactome map

Our approach to defining the physical Tax interactome began with the selective isolation of Tax-containing multi-protein complexes from mammalian cells. The isolation of multi-protein complexes was facilitated by the use of affinity tagged Tax protein. The S-Tax-GFP vector expresses full length TAX protein fused to amino-terminal His_6 _and S-tags, and carboxyl-terminal GFP protein. A critical property in such a system is the recapitulation of Tax-associated activity in the fusion protein. We have previously demonstrated that the expressed S-Tax fusion protein is fully functional when compared to wild type Tax protein [18,19]. The S-Tax-GFP vector was transiently transfected into 293T cells, and the expression of GFP used to assess correct cellular localization and to monitor the transfection efficiency. The S-Tax-GFP protein was purified on S-agarose beads and incubated with Jurkat nuclear extracts. We used the nuclear extracts to increase the relative abundance of Tax binding proteins to Tax. A series of preliminary experiments were conducted in order to titer the best proportions between nuclear lysate concentration and the amount of Tax such that the Tax protein concentration does not either overwhelm the binding partners or disappear from the complex. In an effort to increase the binding specificity of Tax associated proteins, we pre-incubated the nuclear lysate with the S-agarose beads as a "pre-clear" step. This resulted in a significant reduction of nonspecific protein hits such as HSP's and common nuclear structural proteins like tubulin and actin. The resulting isolated protein complexes were then trypsinized and subjected to LC-MS/MS analysis. When each of the three experimental runs was analyzed individually and then compared, we observed that 86% of the proteins were present on all three runs. The control experiments with the S-GFP protein alone resulted in a list of approximately 25 proteins consisting mainly of HSP's, actin and tubulin. Only 10% of these proteins were shared with the S-Tax-GFP experiments.

One approach to assigning value to any single protein-protein interaction is by determining the strength of interaction. A comparable evaluation in mass spectrometry would be measurements that imply the relative sequence coverage of a particular protein within a complex. The number of peptides with sequence unique to the protein (unique peptides), the sum of the relevant peptide confidence scores (protein score), the percentage of sequence coverage (coverage) and the relative abundance of predicted peptides from a protein (emPAI) were used for ranking the Tax-binding protein identities. Such confidence values would be directly influenced by the amount of measurable protein and indirectly influenced by strength of binding. Thus, we combined the data, in which the Tax interactome was analyzed as described above, from three separate experimental runs into one data set. Each of the LC-MS/MS runs contained approximately 23,000 scans. The top 5 protein "hits" as determined via multiple measures of confidence are shown in table 2. This analysis resulted in the identification of a novel interaction between Tax and DNA-PK. We note that one possible explanation for our approach uniquely identifying DNA-PK is the enrichment of nuclear proteins in the binding reaction.

**Table 2:** Tax binding proteins sorted by number of unique peptides

**Protein**	**Unique peptides**	**Protein score**	**Coverage**	**emPAI**
DNA-dependent Protein Kinase	25	1391	9%	0.27
Vimentin	11	1387	44%	7.54
Gamma interferon-inducible protein	19	1116	24%	1.7
PARP	15	1414	34%	1.78
H2A.1	7	569	30%	1.25

### Defining first neighbor interactions of the known Tax-binding proteins

**Figure 1:**
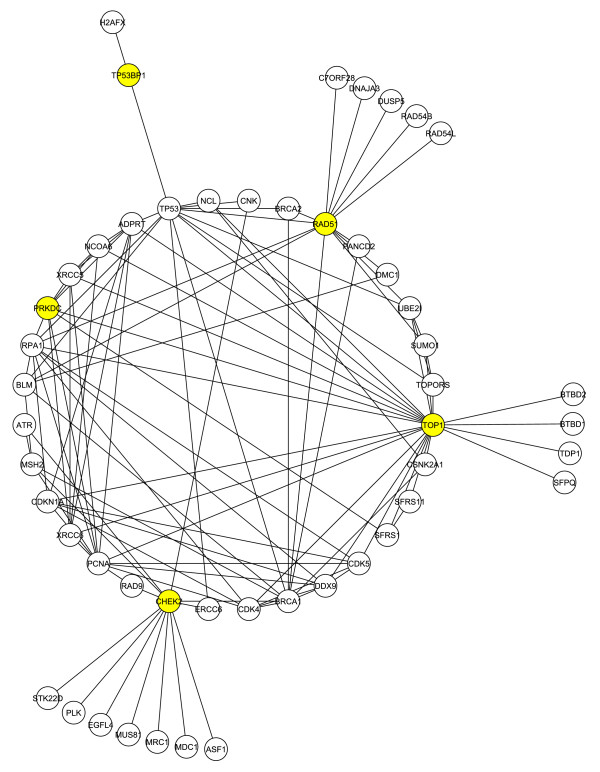
**The G1 first neighborhood network for Rad51, TOP1, Chk2 and 53BP1**. The four initial proteins (yellow) were used to generate a network via interrogation of the Human Protein Reference Database. Protein-protein interactions are indicated by lines. Proteins with two or more shared interactions will form a core. PRKDC (DNA-PK) is also highlighted.

In this section we conducted a query for immediate binding partners of a selected group of known Tax-binding proteins. Our starting group of Tax-binding proteins, Rad51, TOP1, CHEK2 (Chk2), and TP53BP1 (53BP1), known to play a role in the DNA repair response, was referred to as the set C1. The goal was to carefully extend the four protein dataset outward to include the first neighbors of known Tax-binding proteins. We then created a network consisting of the first neighbor interactions of these four proteins with the world of proteins within the HRPD, which we call G1 = 1NN (C1). This sub-network, G1, consists of a set of 50 proteins involved in 112 interactions as shown in figure 1. The G1 sub-network has a diameter of 5, and average path length of 2.7, which are consistent with a small-world network.

Several features in the network G1 and other sub-networks of G1 described below, suggest a significant role for PRKDC(DNA-PKcs). The maximum core (a group of proteins with the most intra-group interactions) of G1 is 6, and DNA-PKcs is a member of the 5-core; the 5-core is a highly interacting group of 12 proteins (DNA-PKcs, TOP1, PCNA, RPA1, DDX9, CDK4, CDKN1A (p21), CDK5, ADPRT (PARP), XRCC5 (Ku70), XRCC6 (Ku86), NCOA6 (TRBP)), all of which are related to the DNA-repair process. Interestingly 6 of these 12 proteins (DNA-PKca, TOP1, DDX9, ADPRT, XRCC5, XRCC6) were also among the Tax-binding proteins observed in the mass spectrometry analysis. We also note that active DNA-PK consists of the catalytic subunit (DNA-PKcs) and the two regulatory subunits (Ku70 and Ku86) each of which is a member of this highly interactive core. Furthermore, DNA-PKcs ranks eighth in degree (the number of interactions) and in the top 30% in two centrality measures (betweenness and closeness).

**Figure 2:**
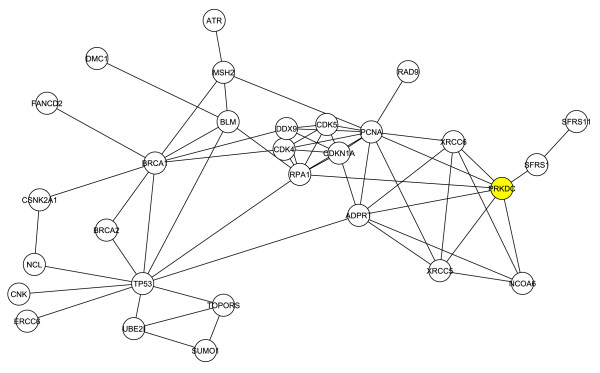
**The largest interacting network remaining in G1 after removal of Rad51, TOP1, Chk2 and 53BP1**. The components that populated the first neighborhood network were depleted of rad51, top1, chk2 and 53bp1. The remaining components with the highest degree of interaction are shown. DNA-PK (PRKDC) is indicated (yellow).

We next considered the structure of the G1 sub-network after the removal of the four initial Tax-binding proteins comprising C1. This would allow for an assessment of the degree and centrality of neighbors without interference from the original four proteins. The largest connected component of the resulting network consisted of 29 proteins and 60 interactions as shown in figure 2. This network has a diameter of 6 and a small average path length of 2.6. In this sub-network, DNA-PKcs is among the top six proteins in degree and betweenness centrality. Thus, the critical role of DNA-PKcs as determined through our clustering process is independent of the presence of the four initial proteins.

**Figure 3:**
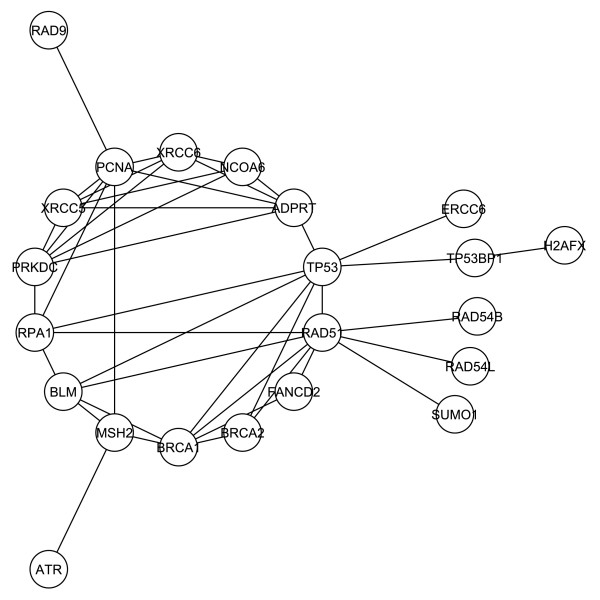
The G1* first neighborhood network restricted to proteins documented to play a role in the DNA-repair response. The components of the entire first neighborhood network were filtered to remove those not known to have a role in the DNA-repair response. The remaining components are displayed to reveal interactions and a central core.

We then created a sub-network of G1 restricted to those involved in DNA repair response, referred to as G1*. Specifically, we removed those proteins that lacked the primary function of DNA repair as listed in the HRPD. This network consisted of 26 proteins and 42 interactions as shown in figure 3. The G1* network has a diameter of 5 and an average path length of 2.5. In this restricted network, DNA-PKcs ranks fourth in degree and ninth in betweenness centrality. The maximum core of this network is the 4-core, which consists of six proteins of which DNA-PKcs is a member (DNA-PKcs, PCNA, PARP, Ku70, Ku86, and TRBP). Thus, DNA-PKcs demonstrates an increased rank when consideration is refocused toward protein interactions involved in DNA damage response.

### Definition of the second neighbors of C1 refined to DNA repair

**Figure 4:**
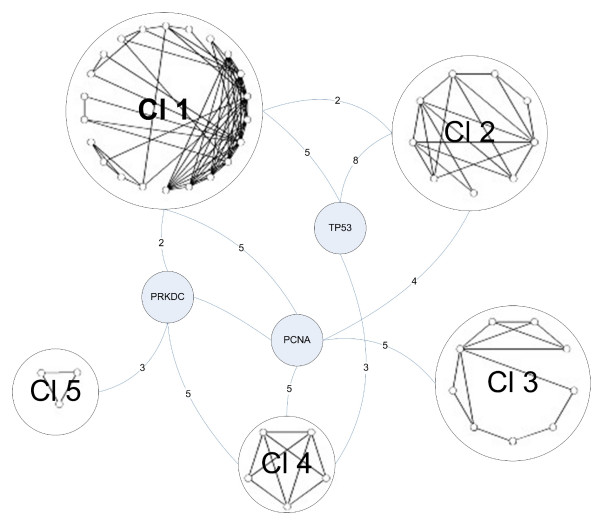
The 3-core representation of the G2* second neighborhood network restricted to DNA damage repair response. Shown is the result of clustering the components of the G2* second neighborhood network arising from the original four Tax binding proteins known to be involved in the cellular DNA damage response. There are five clusters with three bridge proteins; DNA-PK is one of the bridge proteins. For clarity in drawing the network, we do not show edges from these three proteins to the individual proteins in the clusters. The numbers on the edges from these proteins to the clusters count the number of edges from each protein to proteins in each cluster.

In our next exercise, we attempt to assign value to the proteins identified in the prior networks by examining their context in the "larger world" of second neighbors. Our assumption was that key proteins from the first neighbor analysis should retain their central role as defined by interactions in the large second neighbor population. Specifically, in this exercise we first extend the database of Tax-interacting proteins outward to include second neighbor proteins (a protein that binds a protein that is known to bind Tax). We considered the first and second neighborhood of the initial set of proteins in C1, which we refer to as G2 = 2NN (C1). The G2 network consisted of 667 proteins and 3827 interactions. From the proteins in the G2 network, we created a smaller network by restricting to proteins involved in DNA repair, and refer to this sub-network as G2*. There were 114 proteins in G2*. Once this group is developed we use a clustering analysis in an attempt to identify the presumed most critical members of the Tax-interacting world restricted to DNA repair response proteins. The clustering process ranks components of the network based upon the intra-group interactions. We show the 3-core of the G2* network, which consists of 54 proteins, in figure 4. All 3-core proteins will have three or more interactions in order to be included in the network. By application of our clustering approach, we expose the structure of this subnetwork. It consists of five clusters of proteins, with the largest cluster having 22 proteins, and the smallest cluster consisting of 3 proteins. Adding proteins of lower degree clearly generates a larger G2* network, but did not change the integrity of the structure of the network (data not shown). We can also observe from the clustering that three proteins, DNA-PKcs, PCNA, and P53 (TP53) link the various clusters to each other. We call these three proteins "bridges", since they connect the different clusters together. Hence, DNA-PKcs is a bridge protein in this second neighborhood network that links clusters 1, 4, and 5, and is also linked to the bridge protein PCNA.

The five clusters depicted in figure 4, anchored to the three prominent bridge proteins (TP53, PCNA and PRKDC), include proteins that play key roles in DNA repair, stress-induced signaling pathways and cell cycle controls. In general, these proteins are discretely associated with the clusters. For example, Cluster 1 includes four members of the Fanconi anemia complementation group (FANCA, D2, E and G). FANC genes mediate a stress related signaling pathway that allows a normal cell to surmount certain types of damage induced in DNA, principally interstrand crosslinks [20]. In contrast, Cluster 2 includes key genes whose proteins mediate cell cycle arrest in response to genotoxic and other cellular stresses. Thus, if these protein interactions reflect a true subset of the proteins that are directly, or indirectly, affected by Tax-1, then this key viral protein has command over some of the principal cellular stress response pathways that might otherwise inhibit cell growth following HTLV1 infection.

### Endogenous DNA-PK co-precipitates with affinity isolated Tax

**Figure 5:**
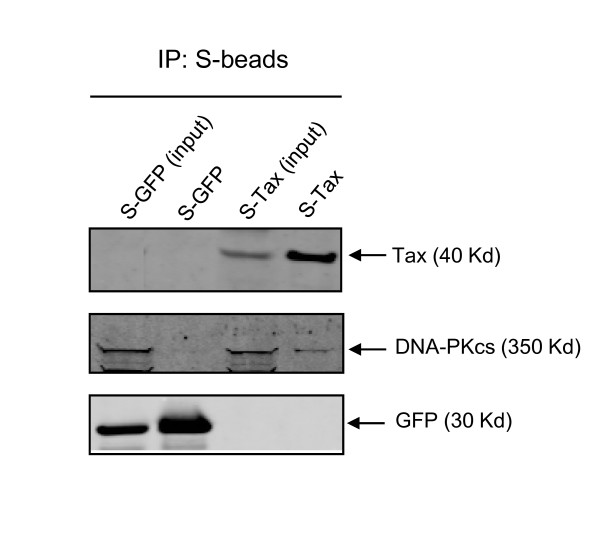
**HTLV-1 Tax binds to DNA-PKcs**. The fusion proteins S-Tax and S-GFP were isolated from 293T cells as described and analyzed for co-precipitation with DNA-PKcs. Shown is the pre-isolated total cell extract (input) for S-GFP (lane 1) and S-Tax (lane 3). Also shown is the affinity purified protein complexes for S-GFP (lane 2) and S-Tax (lane 4). Experimental normalization was achieved by using equal amounts of purified protein.

As a final verification of the binding between Tax and DNA-PKcs, we performed an affinity pull-down of endogenous cellular Tax protein complexes. In this study, we expressed either S-Tax or S-GFP via transient transfection of 293T cells and normalized for S-fusion protein amount. The extracts were then isolated by affinity purification of the S peptide and the complexes separated on SDS-PAGE and subjected to immunoblotting with anti-DNA-PKcs. Endogenous DNA-PKcs specifically associates with the Tax containing protein complex and is detected by staining with anti-DNA-PKcs (Figure 5). These results confirm the identification of DNA-PKcs as a Tax-binding protein.

## Discussion

The HTLV-1 Tax protein has been defined by the proteins with which it interacts [21]. Therefore, it stands to reason that defining the functional properties of this protein will require an understanding of which cellular proteins it interacts with. Clearly, uncovering all potential interactions will include those with functional significance. However, determining which interactions support function and which interactions are of no consequence is an obvious and critical question. We have taken the approach that if we assume that Tax impacts the DNA damage repair process, as many studies support, then those interactions that are critical to the DNA damage repair process will hold greater promise of functional significance. Given this hypothesis, we devised a computational biology approach to help define which physical interactions warrant further study.

One of the challenges in computational systems biology is to create a tool to identify functional modules and the interactions among them from large-scale protein interaction networks. There are three major clustering approaches that have been employed to identify functional modules in proteomic networks. The first approach searches for sub-graphs with specified connectivity, called network motifs, and characterizes these as functional modules or parts of them. This approach is not scalable for finding larger clusters in large-scale networks. The second approach, an example of which is work by Bader and Hogue [22], identifies a seed vertex, around which to grow a cluster. The seed vertex is identified by choosing a vertex of largest weight, where the weight of a vertex is a measure of the number of edges that join the neighbors of the vertex, the clustering coefficient. A vertex in the neighborhood of a cluster is added to it as long as its weight is close (within a threshold) to the weight of the seed vertex. Once a cluster has been identified, the procedure is repeated with a vertex of largest weight that currently does not belong to a cluster as the seed vertex. However, our experience comparing this approach with the spectral algorithms we employed in this study indicates that this method is less stable (i.e., the clusters obtained depend strongly on the seed vertices chosen). We used an improved clustering method [23] to reveal proteins that form functional modules, i.e., multiple proteins involved in the same biological function. This approach was used to apply an objective measure to the functional significance of a protein. Specifically we use this to both cluster proteins into specific functional domains as well as to objectively measure each individual protein's value to that functional domain.

When we compared these results to the Tax-binding proteins generated from our physical mapping efforts, DNA-PK was in the top five best represented binding proteins and occupied a top tier ranking via our functional clustering for DNA damage proteins. Clearly, DNA-PK is a critical component in cellular processes that mediate response to damage and thus the fact that our clustering analysis places high value on this protein is as much a validation of the process as it is novel information. However, we began with a network of known Tax-binding proteins and their neighbors and second-neighbors, and DNA-PK was selected, through our functional clustering approach, whereas other equally critical damage response proteins were not. For instance, among the PI3K protein family members ATM and ATR hold positions of prominence in the DNA damage-response arena equal to DNA-PK [24]. In fact, the three proteins are considered redundant in specific pathways and are sometimes able to substitute functionally [25-27]. However, neither of the other two proteins was reflected in the upper tier interactions when using the Tax-designated protein networks. Furthermore, ATM and ATR were not found among the list of Tax-binding proteins identified in the physical isolation of Tax complexes, again verifying the novelty of the DNA-PK finding.

This is not the first time that DNA-PK has been targeted as a cellular protein through which Tax might mediate genomic instability [28]. It is clear that DNA-PK is known to mediate functions associated with reported Tax activities. Specifically, Tax has been shown to cause constitutive activation of Chk2, a downstream target of DNA-PK [19]. DNA-PK can phosphorylate the tumor suppressor p53 at S15 and S37 [29] whereas Tax expression results in phosphorylation at S15 and S392 [30,31]. In addition, we have recently shown that Tax interaction with DNA-PK results in saturation of the damage response (manuscript submitted). Thus, the Tax-DNA-PK interaction satisfies several previous observations regarding Tax function and provides a unifying model for all of these activities. Thus, although Van et al. [32] demonstrated that the Tax-p53 nexus was intact in a DNA-PK knock-out line, it may well be worth examining this protein as a mediator of other Tax activities.

Clearly HTLV-1 Tax presents a biological model for an interesting protein with an overwhelming amount of associated published literature. A recent review by Boxus et al highlights this complexity and presents an exhaustive compilation of all known Tax-interacting proteins [33]. The growth in the Tax knowledge base requires constant surveillance and verification if this body of work is to be useful in understanding how Tax functions. Additionally, as proteomic techniques continue to mature, the data generated in experimental studies is increasing exponentially. We have described a parallel process for combining *in silico *analysis with experimental proteomic analysis so that information gained in each process facilitates data mining of the orthogonal process. Further building of the Tax interactome should reveal other critical proteins that play key roles in mediating the biologically significant Tax functions within the host cell.

## Methods

### Cell culture and transfection

293T cells were maintained at 37°C in a humidified atmosphere of 5% CO_2 _in air, in Iscove's modified Dulbecco's medium supplemented with 10% fetal bovine serum and 1% penicillin-streptomycin. Transient transfections were performed by standard calcium phosphate precipitation. The plasmid used for expression of S-Tax-GFP has been described previously [18]. For expression of S-Tax and S-GFP the *tax *or *EGFP *open reading frame was inserted into the SmaI site of *pTriEx4-Neo *(Novagen, Madison, WI). Cells were plated in 150-mm plates at 4 × 10^6 ^cells per plate. The following day, 20 μg of plasmid DNA in 2 M CaCl_2 _and 2X HBS were added drop wise to cells in fresh medium. Cells were incubated at 37°C for 5 h and fresh medium was added. The cells were harvested 48 h later.

### Purification of S-fusion proteins

S-Tax-GFP, S-Tax, or S-GFP protein was isolated following a single wash with 1X PBS, in 500 μl M-Per mammalian protein extraction reagent (Pierce, Rockford, IL) supplemented with protease inhibitor cocktail (Roche, Palo Alto, CA) and immediately frozen at -80°C. The cell lysate (2.5 mL) was incubated with 200 μl bed volume of S-protein™ agarose (Novagen, Madison, WI) for 30 min at room temperature as per manufacturer's suggestion. The bound S-tagged protein was then washed 3 times with 1 mL Bind/Wash Buffer (20 mM Tris-HCl pH 7.5, 150 mM NaCl, 0.1% TritonX-100).

### Isolation of Tax-complexes

Freshly prepared S-Tax-GFP or S-GFP beads were washed 3× in incubation buffer (25 mM HEPES, pH 7.5, 150 mM NaCl, 1% NP-40, 10 mM MgCl2, 1 mM EDTA, 1% glycerol) and placed on ice. A working stock of Jurkat nuclear lysate (Active Motif, Carlsbad CA) was prepared by diluting 25 μg lysate to a total volume of 75 μL in incubation buffer. The lysate was pre-cleared by adding 30 μL of S-bead slurry and incubating on ice for 30 minutes with occasional mixing. The pre-cleared slurry was spun down at 2000 g for 3 minutes and the lysate (70 μL) transferred to a fresh 0.5 ml tube containing 10 μL of the S-Tax-GFP or S-GFP protein bound to beads. This slurry was incubated at 4°C for 60 minutes on a shaker. The beads were centrifuged at 2000 g for 3 minutes, lysate removed, and beads washed 1× with 250 μL incubation buffer followed by 4 washes with 250 μL ice cold PBS.

### Isolation of endogenous DNA-PK-Tax protein complex

In some cases, S-Tax or S-GFP expression plasmids were transfected into 293T and protein complexes isolated as described above from a single T75 flask. In these experiments no nuclear extracts were added. The protein lysates were subjected to purification on S-beads, 50 μL of sample loading buffer (Bio-Rad, Hercules, CA) with β-mercaptoethanol was added to the S-bead pellet and boiled for 10 min. The whole protein sample that was bound to the S-bead was separated by 4–12% SDS-PAGE and analyzed by Western Blot as described below.

### LC-MS/MS of protein complexes

S-Tax-GFP or S-GFP beads were washed 3X with ice cold 50 mM ammonium bicarbonate, pH 8 and subsequently resuspended in 50 μL of 50 mM ammonium bicarbonate, 10% acetonitrile containing 3.12 ng/μL sequencing grade modified trypsin (Promega Corp., Madison, WI). The digest was incubated for 6 hours at 37°C with occasional mixing, transferred to a 0.2 μm centrifuge tube filter and spun at 5000 rpm for 3 minutes. The flow through was recovered and peptides dried in a speed vac. Digests were resuspended in 20 μl Buffer A (5% Acetonitrile, 0.1% Formic Acid, 0.005% heptafluorobutyric acid) and 10 μl were loaded onto a 12-cm × 0.075 mm fused silica capillary column packed with 5 μM diameter C-18 beads (The Nest Group, Southborough, MA) using a N2 pressure vessel at 1100 psi. Peptides were eluted over 300 minutes, by applying a 0–80% linear gradient of Buffer B (95% Acetonitrile, 0.1% Formic Acid, 0.005% HFBA) at a flow rate of 150 μl/min with a pre-column flow splitter resulting in a final flow rate of ~200 nl/min directly into the source. A LTQ™ Linear Ion Trap (ThermoFinnigan, San Jose, CA) was run in an automated collection mode with an instrument method composed of a single segment and 5 data-dependent scan events with a full MS scan followed by 4 MS/MS scans of the highest intensity ions. Normalized collision energy was set at 28%, activation Q was 0.250 with minimum full scan signal intensity at 1 × 10^5 ^with no minimum MS^2 ^intensity specified. Dynamic exclusion was turned on utilizing a three minute repeat count of 2 with the mass width set at 1.0 m/z. Protein searches were performed with MASCOT version 2.2.0 v (Matrix Sciences, London GB) using the SwissProt version 51.3 database. Parent ion mass tolerance was set at 1.5 and MS/MS tolerance 0.5 Da.

### Western analysis

Total protein concentrations were determined by Protein Assay (Bio-Rad, Hercules, CA). An equal volume of sample loading buffer (Bio-Rad, Hercules, CA) with β-mercaptoethanol was added to the lysate and boiled for 5 min. Samples were normalized to total protein and separated through a 10% SDS-polyacrylamide gel. The proteins were transferred onto Immobilon-P (Millipore, Billerica, MA) membrane using a Trans-blot SD semi-dry transfer cell (Bio-Rad, Hercules, CA) at 400 mA for 50 min. Following blocking in 5% non-fat milk in PBS/0.1% Tween-20, blots were incubated in primary antibody overnight, followed by 1 h incubation in secondary horseradish-peroxidase conjugated anti-mouse or anti-rabbit antibody (Bio-Rad, Hercules, CA). Immunoreactivity was detected via Immunstar enhanced chemiluminescence protein detection (Bio-Rad, Hercules, CA). The following primary antibodies were used in the analysis: mouse monoclonal antibody of DNA-PKcs (Upstate), 1:1000; rabbit polyclonal antibody of Tax, 1:5000; mouse monoclonal antibody of GFP (Santa Cruz), 1: 2000.

### Sources of data for *in silico *analysis

Interaction data were gathered from three types of information sources: manual extraction from Pubmed, laboratory derived physical interactions, and protein interaction databases. In the first database source, the information was extracted by manually searching the Pubmed literature to obtain a list of known Tax binding proteins. The criterion for acceptance in this group was physical verification of binding in the referenced publication. For the second database source, the physical interactions utilized in this study were all derived from the experimental efforts described elsewhere in this article. For the final database source, we queried a human protein interaction database; The Human Protein Reference Database (HPRD) [34]. The HPRD  contains interactions of proteins in the human proteome manually extracted from the literature by expert biologists who read, interpret and analyze the published data.

### Terms and definitions for *in silico *analysis

For our topological studies of interaction networks, we utilized a novel overlapping clustering approach [23] that exposes the modular structure of the network. We define bridges as proteins that belong to multiple clusters due to the overlap among them. We also employed centrality measures of networks known as betweenness and closeness. To define these measures, first we need to define some network concepts. The distance of a protein *v *from another protein *w *is the number of edges in a shortest path between them. The diameter of a network is the maximum distance between any pair of vertices. The average path length of a network is the average distance over all pairs of vertices. The closeness centrality measure for a protein, *v*, is the reciprocal of the sum of the distances of *v *to all other proteins in the network.

The dependence of a protein *s *on a protein *v *is the sum over all proteins t in the network of the ratio of the number of distinct shortest paths between proteins *s *and *t *that includes *v *as an intermediate vertex, and the number of distinct shortest paths between *s *and *t*. The betweenness value of a protein *v *is the sum of the dependence values of all proteins *s *on the protein *v*. This is equivalent to the following equation for betweenness.

$$B(v)=\sum_{\begin{array}{c} s\in v\\ s\neq v \end{array}}\sum_{\begin{array}{c} t\in v\\ t\neq s,\\ t\neq v \end{array}}\frac{\sigma_{st}(v)}{\sigma_{st}}$$

Here V is the set of proteins in the network. The numerator in the fraction shows the number of distinct shortest paths joining s and t on which v is an intermediate vertex; the denominator is the number of distinct shortest paths joining s and t. Further details on centrality measures are available in [35].

As in earlier work [36], we define *hubs *as all proteins that are ranked in the top 20% with respect to degree in the network (the number of interactions a protein is involved in). Similarly *bottlenecks *are all the proteins that are ranked in the top 20% of betweenness values. To calculate betweenness values for proteins, we used an algorithm provided by Yu et al. [37].

In the clustering approach to be described next, we use the concept of a *k-core *of a graph. The *k*-core of a graph is obtained by repeatedly deleting all vertices which are joined to the vertices remaining in the graph by fewer than *k *edges. This procedure begins by deleting all vertices whose degree is less than *k*. The deletion of such vertices could decrease the degrees of the remaining vertices. If some of these vertices have degrees less than *k*, they would be deleted as well. This process is repeated until the subgraph that remains has every vertex with degree at least *k*; this subgraph is the k-core of the graph. All the deleted vertices belong to the *(k-1)-shell*. Computing the k-core of a graph helps with denoising the interaction network by removing many false positives, and also reduces the initial size of the network to be clustered. The deleted vertices will be added to the clustering obtained in a subsequent step.

### Spectral clustering and modules identification

We now summarize the technique we used for clustering the protein interaction networks [23]. The protein interaction network is represented by a graph G = (V, E), with the proteins constituting a set of proteins V, and interactions constituting the set of edges E. We obtain clusters in the interaction network by identifying a number of subgraphs of G that have a relatively large number of edges joining vertices in each subgraph and fewer edges to vertices outside the subgraph. We permit these clusters to overlap (have some vertices in common), since proteins have multiple functions and could be involved in more than one biological process.

The details of the clustering algorithm will be described elsewhere, but here we provide an overview. Clusters are obtained by dividing a subgraph at each step into two subgraphs based on the ratio of the number of edges that join vertices in the subgraph to the total number of edges, a measure called the *cohesion *of the subgraph. Given the initial graph G, we recursively split it into subgraphs until the value of cohesion of a subgraph is above a threshold value, or the subgraph has number of vertices fewer than a threshold size. We have used a spectral algorithm that uses the components of an eigenvector of the Laplacian matrix of the graph to divide each subgraph into two. Once the eigenvector is computed (its components correspond to the vertices of the graph), those vertices whose component values are below some specified value are included in one subgraph and the others belong to the second subgraph. The choice of the value where the split should be made is based on computing the cohesion.

We have found that the overall clustering approach described above needed to be adapted to protein interaction networks, which are small-world and modified power-law networks. Initially we decompose the vertices of the network into three sets; hubs or high degree vertices (those in the top 20% of the degrees); low-shell vertices (vertices not in the 3-core of the network); and the residual sub-network, which forms a 3-core of the network from which the hubs have been removed. We call the last subnetwork as the local network. We have found it advantageous to cluster the local and hub sub-networks separately using the spectral clustering method described above. The clusters from both sub-networks are then merged together if a large number of edges join clusters from the two networks. We check to see if nodes that belong to a cluster are significantly connected to other clusters, and if so, they are included in such clusters as well. The statistical significance of the connections is computed using a *p*-value based on the hypergeometric distribution. Finally, the low-shell nodes are added to clusters; each such node could be added to none, one, or more than one cluster, based on whether it has a statistically significant number of connections to the clusters that have been found. If a node belongs to three or more clusters, we call it a bridge node.

## Competing interests

The authors declare that they have no competing interests.

## Authors' contributions

ER performed the computational experiments on the interaction networks. MW performed all mass spectrometry analysis. XG and SD conducted the Tax-DNA-PKcs binding experiments. AS contributed to the compilation of Tax binding proteins. MV was responsible for study design and interpretation of results. CO was involved in aspects of study design and manuscript preparation. AP designed the network algorithms and helped with the writing. OJ designed the study, interpreted results and contributed to manuscript preparation.
